# Modeling and Mechanistic Analysis of Molten Pool Evolution and Energy Synergy in Laser–Cold Metal Transfer Hybrid Additive Manufacturing of 316L Stainless Steel

**DOI:** 10.3390/ma19020292

**Published:** 2026-01-11

**Authors:** Jun Deng, Chen Yan, Xuefei Cui, Chuang Wei, Ji Chen

**Affiliations:** 1 China Petroleum Pipeline Research Institute Co., Ltd., Langfang 065000, China; 2 National Engineering Research Center for Oil and Gas Pipeline Transportation Safety, Langfang 065000, China; 3 School of Materials Science & Engineering, Welding Institute, Shandong University, Jinan 250061, China

**Keywords:** laser–CMT hybrid additive manufacturing, spatial orientation, multi-layer deposition, molten pool behavior, computational modeling

## Abstract

**Highlights:**

**What are the main findings?**

- Developed a self-adaptive CMT arc heat source for laser–CMT hybrid modeling.
- Provided insight into orientation-governed thermal and flow field mechanisms.
- Revealed orientation-dependent molten pool flow and temperature evolution.

**What are the implications of the main findings?**

- Enables higher simulation fidelity in predicting complex multiphysics interactions for hybrid directed energy deposition.
- Provides practical guidelines for optimizing process parameters and scanning strategies to control deposition quality.

**Abstract:**

The present work uses numerical methods to explore the impact of spatial orientation on the behavior of molten pool and thermal responses during the laser–Cold Metal Transfer (CMT) hybrid additive manufacturing of metallic cladding layers. Based on the traditional double-ellipsoidal heat source model, an adaptive CMT arc heat source model was developed and optimized using experimentally calibrated parameters to accurately represent the coupled energy distribution of the laser and CMT arc. The improved model was employed to simulate temperature and velocity fields under horizontal, transverse, vertical-up, and vertical-down orientations. The results revealed that variations in gravity direction had a limited effect on the overall molten pool morphology due to the dominant role of vapor recoil pressure, while significantly influencing the local convection patterns and temperature gradients. The simulations further demonstrated the formation of keyholes, dual-vortex flow structures, and Marangoni-driven circulation within the molten pool, as well as the redistribution of molten metal under different orientations. In multi-layer deposition simulations, optimized heat input effectively mitigated excessive thermal stresses, ensured uniform interlayer bonding, and maintained high forming accuracy. This work establishes a comprehensive numerical framework for analyzing orientation-dependent heat and mass transfer mechanisms and provides a solid foundation for the adaptive control and optimization of laser–CMT hybrid additive manufacturing processes.

## 1. Introduction

The field of metallic additive manufacturing (AM) has advanced quickly and is becoming vital for producing complex components used in aerospace, marine, and chemical applications. Among the different AM methods, laser–arc hybrid additive manufacturing (LAHAM) stands out for integrating the strengths of laser and arc heat sources. The laser irradiation stabilizes the arc, while the arc reduces the substrate’s laser reflectivity and enhances laser energy absorption, leading to higher deposition efficiency, improved joint quality, and better process adaptability [1,2,3].

Prior investigations into laser–arc hybrid welding have confirmed these synergistic effects, showing reduced spatter, improved arc stability, and enhanced molten pool flow behavior. Extending this concept to AM, LAHAM integrates the high precision and low dilution characteristics of laser processes with the high deposition rate and cost efficiency of arc-based techniques. This combined approach offers the advantages of high efficiency and improved microstructural refinement, with its effectiveness demonstrated in multiple recent investigations [4,5,6,7]. Zhang et al. employed a laser–tungsten inert gas hybrid heat source to fabricate multi-layer 316L stainless steel walls, finding that the laser addition reduced heat input by 26.4%, refined the microstructure, and improved hardness and tensile strength compared with conventional TIG-based AM [8]. Gong et al. investigated the effect of laser power on molten pool behavior and texture evolution during laser–arc hybrid AM of 316L steel, revealing a transition from conductive to keyhole mode as laser power increased from 500 W to 2000 W, which enhanced cube texture formation and tensile strength [9]. Pardal et al. demonstrated that laser assistance in a conduction-mode laser–Cold Metal Transfer (CMT) hybrid process improved deposition stability and efficiency by up to 82% for Ti-6Al-4V alloy [10]. Several experimental studies have explored hybrid strategies for aluminum alloys (laser–CMT and laser–plasma hybrid), showing that appropriate laser power could reduce porosity and eliminate columnar grains, improving tensile properties [11,12,13]. Work on droplet transfer and impact behavior under hybrid conditions showed a strong dependence on electromagnetic forces and gravity orientation, indicating that spatial position altered droplet impingement and molten pool response [14,15]. Recent experimental advances and method papers include hybrid wire–laser or plasma–laser wire deposition and asynchronous hybrid direct energy deposition strategies, expanding practical process windows for 316L and other alloys [16,17].

Although experimental studies have uncovered key macroscopic characteristics of laser–arc hybrid processes, they are unable to provide detailed quantitative data on internal transport phenomena. Critical parameters like transient temperature variations, flow velocity, and localized energy transfer are difficult to measure experimentally. Consequently, numerical simulations have been widely used to explore heat transfer, fluid dynamics, keyhole behavior, and solidification processes, delivering in-depth understanding of mechanisms that experiments cannot directly observe. Multiphysics CFD and finite element models that couple VOF surface tracking, Marangoni convection, recoil pressure, and coupled heat sources have been used to predict molten pool shape, temperature cycles, and solidification conditions [18,19]. Several recent LAHAM-specific simulation studies implemented composite heat-source models (Gaussian laser + double-ellipsoid arc) and validated predicted molten pool geometry and thermal cycles against experiments. Wang et al. developed a three-dimensional (3D) dual-heat-source model and demonstrated good agreement of predicted molten pool shape, HAZ and thermal cycles with experimental measurements; parametric studies identified strong sensitivity of molten pool geometry to laser power and scan speed [20]. Jin et al. performed molten pool finite element analysis for aluminum and titanium hybrid welding and AM, investigating conduction vs. keyhole regimes and their influence on remelting and solidification [21]. Although LAHAM has demonstrated significant advantages in improving deposition quality and mechanical performance, several key issues remain unresolved. Mechanistic insights into the transient coupling of temperature, flow, and energy transfer within the molten pool are still limited, particularly under varying spatial orientations where gravity direction changes. Moreover, current heat-source models are mostly parameter-fixed and cannot self-adapt to variations in wire feed speed, scanning speed, or deposition posture, while the interaction between dynamic laser absorption and keyhole wall temperature is still not fully captured in simulations.

This study overcomes these limitations by combining high-speed imaging techniques with a three-dimensional coupled model that includes dynamic laser absorption and adaptive dual heat-source parameters. The proposed adaptive double-ellipsoidal CMT arc model coupled with temperature-dependent laser absorptivity improves prediction accuracy by dynamically adjusting heat source parameters according to process conditions. Compared to fixed-parameter Gaussian + double-ellipsoid or VOF-coupled CFD models, this approach more effectively represents transient thermal-fluid behavior and keyhole dynamics with improved computational efficiency. This work systematically explored the behavior of the molten pool spatially in laser–CMT hybrid additive manufacturing of 316L stainless steel, focusing on the impacts of energy coupling and gravity orientation effects on molten pool morphology, heat transfer, and fluid flow. The results provide fundamental insights to enhance the optimization of hybrid AM techniques for achieving high precision and efficiency.

## 2. Experimental Design and Numerical Modeling

### 2.1. Experimental Setup

**Figure 1:**
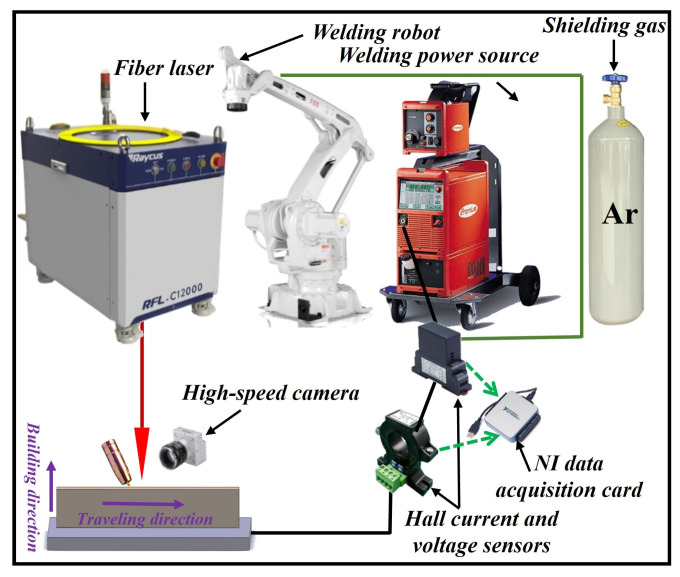
Schematic diagram of laser–CMT arc hybrid additive manufacturing experiment.

A laser–CMT hybrid additive manufacturing system was developed, as illustrated in Figure 1. The setup consisted of four primarily components: a laser control system, an arc cladding system, an electrical signal monitoring system, and an imaging device. The substrate material selected was a 316L stainless steel plate measuring 250 mm × 50 mm × 5 mm, and the filler wire was ER316L stainless steel with a diameter of 1.2 mm. The detailed chemical composition of the wire can be found in ref. [22]. Experiments were performed using the CMT mode, with the process parameters detailed in Table 1. A shielding gas mixture of 98% Ar and 2% O_2_ was delivered at a flow rate of 25 L/min, and the stick-out distance was kept at 10 mm.

### 2.2. Numerical Model

#### 2.2.1. Base Assumptions

To developed the numerical model for heat-mass transfer during laser–CMT hybrid additive manufacturing, the following assumptions were adopted:

(1) The molten metal was modeled as an incompressible Newtonian fluid, with laminar flow conditions assumed.
(2) The filler wire and substrate were considered to have the same chemical composition and thermophysical properties.
(3) Effects of the shielding gas on the molten pool and keyhole formation were neglected.
(4) Metal evaporation and spatter losses were not taken into account.

**Figure 2:**
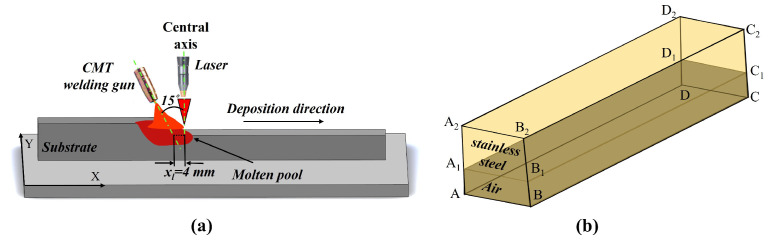
Laser–CMT arc hybrid additive manufacturing: (**a**) schematic diagram of deposition process, (**b**) geometric model.

The numerical model was depicted in Figure 2a,b presents the simplified 3D domain representing the composite heat source. Owing to geometric symmetry about the weld centerline, only half of the domain was modeled to improve computational efficiency. The computational domain had dimensions of 70 mm × 14 mm × 17 mm, comprising a molten metal zone 5 mm high and an air zone 12 mm high. The mesh size was 0.25 mm within the metallic zone and 0.3 mm within the air zone. The angle between the welding gun and the laser heat source was set to 15°, and the distance between the electric arc axis and the laser beam axis was $x_{l}$ (4 mm in this study).

To simulate the heat transfer and fluid flow during the composite CMT arc additive manufacturing process, the Flow-3D 9.3 software was employed, utilizing the three-step Volume of Fluid (VOF) method to track and handle the free surface dynamics. The iterative solution scheme involved solving the pressure correction equation using the Successive Over-Relaxation (SOR) method to satisfy mass conservation, followed by an implicit solution of the energy conservation equation.

#### 2.2.2. Governing Equations

A three-dimensional coupled thermo–fluid model was established to analyze the transient behavior of the molten pool in laser–CMT hybrid additive manufacturing. This model combined the conservation equations of mass, momentum, and energy with the Volume of Fluid (VOF) technique for free-surface tracking.

Mass conservation equation

(1) $$\rho V_{droplet}=m_{s}$$

(2) $$V_{droplet}=\pi v_{wire}r_{wire}^{2}\Delta t$$

(3) $$\Delta t=\mathrm{mod}\left(t_{welding}/t_{droplet}\right)$$

where ρ is the mass density, $V_{droplet}$ is the droplet volume, $m_{s}$ is the droplet-induced mass source term, $v_{wire}$ is the wire feeding speed, $r_{wire}$ is the wire radius, $t_{welding}$ is the welding time, $t_{droplet}$ is the droplet transfer time (equal to 0.0148 ms at 210 A). It means that a droplet will detach from the wire tip when Δt=0 (Equation (3)). At this time, an artificial lateral shear force is applied to forcibly detach the droplet from the wire tip, after which a new droplet begins to grow in the subsequent time step. When Δt≠0, the droplet undergoes growth.

Momentum conservation equation

(4) $$\rho \left[\frac{\partial \vec{V}}{\partial t}+\left(\vec{V}\cdot \nabla \right)\vec{V}\right]=-\nabla P+v\nabla^{2}\vec{V}-K\vec{V}+S_{D}$$

where P is the pressure, v is the dynamic viscosity, K is the permeability, $S_{D}$ is the momentum source term, which includes multiple body forces:

(1) Electromagnetic force (r ≠ 0)

(5) $$\begin{array}{l} F_{x}=-\frac{\mu_{m}I^{2}}{4\pi^{2}\sigma_{j}^{2}r}\exp\left(-\frac{r^{2}}{2\sigma_{j}^{2}}\right)\left[1-\exp\left(-\frac{r^{2}}{2\sigma_{j}^{2}}\right)\right]{\left(1-\frac{z}{d}\right)}^{2}\frac{\left(x-v_{w}t\right)}{r}\\ F_{y}=-\frac{\mu_{m}I^{2}}{4\pi^{2}\sigma_{j}^{2}r}\exp\left(-\frac{r^{2}}{2\sigma_{j}^{2}}\right)\left[1-\exp\left(-\frac{r^{2}}{2\sigma_{j}^{2}}\right)\right]{\left(1-\frac{z}{d}\right)}^{2}\frac{y}{r}\\ F_{z}=\frac{\mu_{m}I^{2}}{4\pi^{2}dr^{2}}{\left[1-\exp\left(-\frac{r^{2}}{2\sigma {\left(t\right)}_{j}^{2}}\right)\right]}^{2}{\left(1-\frac{z}{d}\right)}^{2} \end{array}$$

where I is the welding current, $\sigma_{j}$ is the effective radius, $\mu_{m}$ is the magnetic permeability.

(2) Arc pressure

The pressure field followed a double-elliptic distribution to represent front and rear arc regions accurately:

(6) $$\begin{array}{l} p_{af}\left(x,y\right)=C\frac{3\mu_{m}I^{2}}{2\pi^{2}\left(a_{j1}+a_{j2}\right)b_{j}}\exp\left[-\frac{3{\left(x-v_{w}t\right)}^{2}}{a_{j1}^{2}}-\frac{3{\left(y-0.5h_{w}\right)}^{2}}{b_{j}^{2}}\right],x\geq 0\\ p_{ar}\left(x,y\right)=C\frac{3\mu_{m}I^{2}}{2\pi^{2}\left(a_{j1}+a_{j2}\right)b_{j}}\exp\left[-\frac{3{\left(x-v_{w}t\right)}^{2}}{a_{j2}^{2}}-\frac{3{\left(y-0.5h_{w}\right)}^{2}}{b_{j}^{2}}\right],x<0 \end{array}$$

where C is the correction factor, $a_{j1}$, $a_{j2}$, and $b_{j}$ are the current distribution parameters, $p_{af}$ is the front half arc pressure, $h_{w}$ is the plate width, $p_{ar}$ is the rear half arc pressure.

(3) Recoil pressure

(7) $$P_{R}=0.54P_{0}\exp\left(L_{v}\frac{T-T_{b}}{RTT_{b}}\right)$$

where $P_{0}$ is the ambient pressure, $L_{v}$ is the latent heat of vaporization, T is the local surface temperature, $T_{b}$ is the boiling point, R is the gas constant.

(4) Surface tension

(8) $$P_{\sigma }=\sigma \left(\frac{1}{R_{1}}+\frac{1}{R_{1}}\right)$$

(9) σ=2.683−0.0004⋅T

(5) Buoyancy

(10) $$F_{f}=\rho g\beta \Delta T$$

where g is the gravitational acceleration.

(6) Vapor shear stress

(11) $$\tau_{w}=\frac{1}{8}f\rho V^{2}$$

Energy conservation equation

(12) $$\rho \left[\frac{\partial \left(h\right)}{\partial t}+\vec{V}\cdot \nabla h\right]=\nabla \cdot \left(\lambda \nabla T\right)+q_{A}+q_{D}+q_{L}$$

where h is the specific enthalpy, ΔT is the temperature difference, λ is the thermal conductivity, $q_{A}$ is the arc heat, $q_{D}$ is the droplet heat, $q_{L}$ is the evaporation heat loss.

VOF equation

(13) $$\frac{\partial F}{\partial t}+\nabla \cdot \left(\vec{V}F\right)=0$$

where F is the volume fraction.

#### 2.2.3. CMT Heat Source Model

A double-ellipsoidal heat source model was adopted to effectively represent the heat distribution during the CMT arc additive process [23]:

(14) $$\begin{array}{l} q_{f}=\frac{6\sqrt{3}f_{f}Q_{A}}{\pi^{2}a_{f}b_{h}c_{h}\sqrt{\pi }}\exp\left(-\frac{3{\left(x-v_{w}t-x_{l}\right)}^{2}}{a_{f}^{2}}-\frac{3{\left(y-0.5h_{w}\right)}^{2}}{b_{h}^{2}}-\frac{3z^{2}}{c_{h}^{2}}\right)\;\;x\geq 0\\ q_{r}=\frac{6\sqrt{3}f_{r}Q_{A}}{\pi^{2}a_{r}b_{h}c_{h}\sqrt{\pi }}\exp\left(-\frac{3{\left(x-v_{w}t-x_{l}\right)}^{2}}{a_{r}^{2}}-\frac{3{\left(y-0.5h_{w}\right)}^{2}}{b_{h}^{2}}-\frac{3z^{2}}{c_{h}^{2}}\right)\;\;x\leq 0 \end{array}$$

(15) $$Q_{A}=\eta UI-Q_{D}$$

(16) $$Q_{D}=\rho c_{p}\Delta T\left(\pi v_{wire}r_{wire}^{2}\right)$$

where $Q_{A}$ is the total arc heat, $a_{f}$, $a_{r}$, $b_{h}$, and $c_{h}$ are the heat distribution parameters, $f_{f}$ is the heat fraction distributed in the front ellipsoid, $f_{r}$ is the heat fractions distributed in the rear ellipsoid, ρ is the mass density, and $c_{p}$ is the specific heat.

Conventional double-ellipsoidal models use fixed parameters, restricting their effectiveness when process conditions such as wire feed speed, scanning velocity, or deposition orientation change dynamically. To overcome this limitation, an adaptive double-ellipsoidal model was introduced, allowing heat source parameters to vary according to process variables based on experimental data fitting. Such efforts align with recent advances in simplified yet physically grounded equivalent-property characterization for stainless-steel components [24], demonstrating ongoing trends toward integrating material and process modeling for improved predictive capability. This adaptive modeling approach lays the groundwork for potential integration into real-time process-control algorithms and digital twin systems, which could significantly enhance industrial applicability by enabling dynamic adjustment of process parameters for optimized additive manufacturing.

A multivariate linear regression expresses these dependencies:

(17) y=X β+ε

In Equation (15),

(18) $$X=\left[1\;\;x_{1}\;\;x_{2}\;\;\cdots \;\;x_{n}\right]$$

(19) $$\beta =\left[\begin{array}{c} \beta_{0}\\ \beta_{1}\\ \vdots \\ \beta_{n} \end{array}\right]$$

In Equations (16) and (17), X contains the independent variables relevant to the process, where $x_{1}$ represents the cladding current, $x_{2}$ is the scanning speed, $x_{3}$ denotes the angle during transverse additive manufacturing, $x_{4}$ corresponds to the angle during downward vertical additive manufacturing, $x_{5}$ corresponds to the angle during upward vertical additive manufacturing. $\beta_{0}$ is the intercept term, while $\beta_{0}$, $\beta_{1}$, $\beta_{2}$,…, $\beta_{n}$ are regression coefficients corresponding to each independent variable.

For instance, the weld width parameter is:

(20) $$b_{h}=\left[1\;\;I\;\;V\;\;\sin\theta_{1}\;\;\sin\theta_{2}\;\;\sin\theta_{3}\right]\left[\begin{array}{c} \beta_{0}\\ \beta_{1}\\ \beta_{2}\\ \beta_{3}\\ \beta_{4}\\ \beta_{5} \end{array}\right]$$

In Equation (18), $\theta_{1}$ is the transverse additive manufacturing angle, ranging from 0° to 90°, $\theta_{2}$ is the vertical-down additive manufacturing angle, ranging from 0° to 90°, and $\theta_{3}$ is the vertical-up additive manufacturing angle, ranging from −90° to 0°.

Experimental data collected under different conditions enables fitting of width-, depth-, and height-related parameters, resulting in a self-adaptive CMT arc heat source model:

(21) $$\left[\begin{array}{c} b_{h1}\\ b_{h2}\\ a_{f}\\ a_{r} \end{array}\right]=\left[\begin{array}{c} 2.60\\ 2.60\\ 5.20\\ 2.60 \end{array}\;\;\;\begin{array}{c} 0.0125\\ 0.0125\\ 0.0250\\ 0.0125 \end{array}\;\;\;\begin{array}{c} -3.30\\ -3.30\\ -6.60\\ -3.30 \end{array}\;\;\;\begin{array}{c} 0.20\\ -0.70\\ -0.50\\ -0.25 \end{array}\;\;\;\begin{array}{c} 0.25\\ 0.25\\ 0.25\\ 0.25 \end{array}\;\;\;\begin{array}{c} 0.60\\ 0.60\\ 1.20\\ 0.60 \end{array}\right]\left[\begin{array}{c} 1\\ I\\ V\\ \sin\theta_{1}\\ \sin\theta_{2}\\ \sin\theta_{3} \end{array}\right]$$

**Figure 3:**
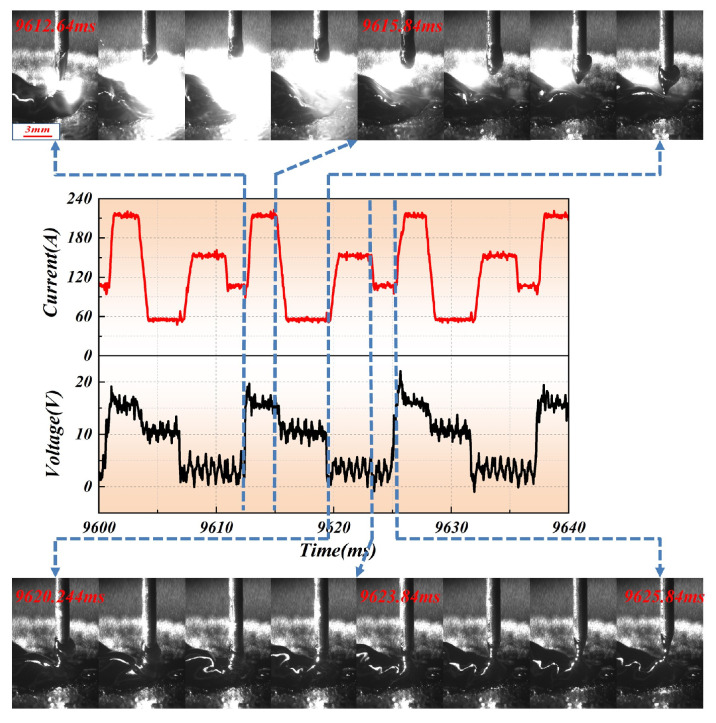
Dynamic behavior changes of arc droplets within one CMT cycle.

Additionally, due to the cyclic waveform of current and voltage during CMT deposition, the heat source parameters are segmented into four stages within one cycle as shown in Figure 3:

(22) $$\left\{\begin{array}{c} I_{1}=210A,\;9612.64\;ms=<t<9615.84\\ I_{2}=55A,\;9615.84\;ms=<t<9620.24\\ I_{3}=155A,\;9620.24\;ms=<t<9623.84\\ I_{4}=105A,\;9623.84\;ms=<t<9625.84 \end{array}\right.$$

In Equation (20), $I_{1}$, $I_{2}$, $I_{3}$ and $I_{4}$ represent the four stages of the CMT cycle: step up ($c_{r1}$), wait ($c_{r2}$), necking ($c_{r3}$), and separation stages ($c_{r4}$), respectively. Consequently, the dynamic changes in penetration-related parameters is described as follows:

(23) $$\left[\begin{array}{c} c_{r1}\\ c_{r2}\\ c_{r3}\\ c_{r4} \end{array}\right]=\left[\begin{array}{c} 1.40\\ 1.40\\ 1.40\\ 1.40 \end{array}\;\;\begin{array}{c} 0.01\\ 0.01\\ 0.01\\ 0.01 \end{array}\;\;\begin{array}{c} -0.20\\ -0.20\\ -0.20\\ -0.20 \end{array}\;\;\begin{array}{c} -0.70\\ -0.70\\ -0.70\\ -0.70 \end{array}\;\;\begin{array}{c} 0.65\\ 0.65\\ 0.65\\ 0.65 \end{array}\;\;\begin{array}{c} -0.90\\ -0.90\\ -0.90\\ -0.90 \end{array}\right]\left[\begin{array}{c} 1\\ I_{1}\\ V\\ \sin\theta_{1}\\ \sin\theta_{2}\\ \sin\theta_{3} \end{array}\right]$$

**Figure 4:**
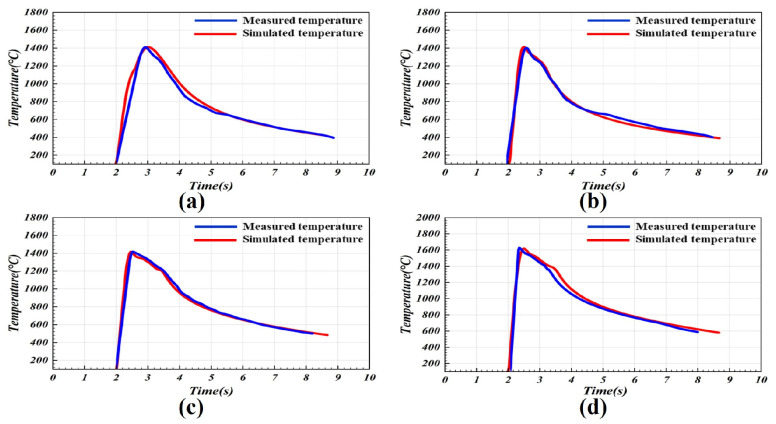
Thermal cycle curve comparison of CMT single-pass deposition simulation and experimental data across different wire feed speeds: (**a**) 4.3 m/min, (**b**) 4.7 m/min, (**c**) 5.3 m/min, and (**d**) 6.0 m/min.

**Figure 5:**
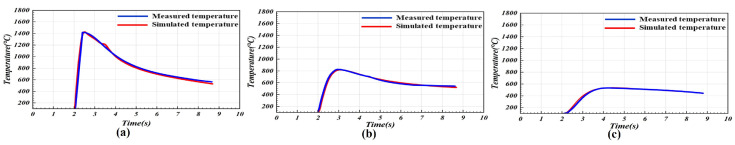
Thermal cycle curve comparison between simulation results and experimental measurements for various deposition layers: (**a**) first layer, (**b**) second layer, (**c**) third layer.

To verify the accuracy of the optimized CMT arc heat source model, the thermal cycles during the additive manufacturing process were recorded using a platinum–rhodium thermocouple and compared with the simulation results, as shown in Figure 4. The experimental and simulated thermal cycle curves exhibited good agreement, indicating that the optimized adaptive CMT arc heat source model accurately reproduced the heat transfer behavior of the substrate during single-track deposition. During the multi-layer additive process, the comparison between measured and simulated thermal cycles was shown in Figure 5. As the number of deposited layers increased, downward heat conduction decreased and the peak temperature gradually declined, which was consistent with the previously obtained conclusions. These results demonstrated that the calculated thermal transfer behavior closely matched the actual experimental observations, further confirming the reliability of the optimized heat source model.

#### 2.2.4. Laser Heat Source Model

The laser input is described by a ray-tracing-based keyhole model:

(24) $$q_{L}\left(x,y,z\right)=\frac{3Q_{L}}{\pi r_{L}^{2}}\exp\left(-3\frac{{\left(x-v_{w}t\right)}^{2}+{\left(y-0.5h_{w}\right)}^{2}}{r_{L}^{2}}\right)$$

(25) $$r_{L}=r_{0}+a\left|z_{0}-z\right|$$

where $Q_{L}$ is the laser power, $r_{0}$ is the focal radius, $z_{0}$ is the focal position, $r_{L}$ is the laser heat source distribution parameter.

Unlike traditional Gaussian or conical models, the ray-tracing algorithm accounts for multiple internal reflections and Fresnel absorption within the keyhole, improving the prediction of deep or narrow vapor cavities. The Fresnel absorption coefficient is [25]:

(26) $$\alpha \left(\theta \right)=1-\frac{1}{2}\left[\frac{1+{\left(1-\varepsilon \cos\theta \right)}^{2}}{1+{\left(1+\varepsilon \cos\theta \right)}^{2}}+\frac{\varepsilon^{2}-2\varepsilon \cos\theta +2\cos^{2}\theta }{\varepsilon^{2}+2\varepsilon \cos\theta +2\cos^{2}\theta }\right]$$

where θ is the angle between incident laser line and normal vector of the small hole wall. In Equation (26), ε is the Fresnel absorption parameter [26]:

(27) $$\varepsilon^{2}=\frac{2\varepsilon_{2}}{\varepsilon_{1}+{\left[\varepsilon_{1}^{2}+{\left(\sigma_{st}/\omega \varepsilon_{0}\right)}^{2}\right]}^{\frac{1}{2}}}$$

where $\varepsilon_{1}$ is the material dielectric constant, $\varepsilon_{2}$ is the plasma dielectric constant, $\varepsilon_{0}$ is the vacuum dielectric constant, ω is the laser angular frequency.

In Equation (27), $\sigma_{st}$ is the material electrical conductivity, and it decreased with increasing temperature. This value affected the laser absorptivity of the metal and their relationship could be expressed as:

(28) $$R_{\rho }=R_{\rho 20}\left(1+K_{\rho }T\right)$$

where $R_{\rho 20}$ is the resistivity at room temperature, taken as 7.5 × 10^−7^ Ω·m, $K_{\rho }$ is the temperature coefficient of resistivity, estimated as 0.004 K^−1^ [27].

The relationship between electrical conductivity and resistivity was given by:

(29) $$\sigma_{st}=\frac{1}{R_{\rho }}$$

**Figure 6:**
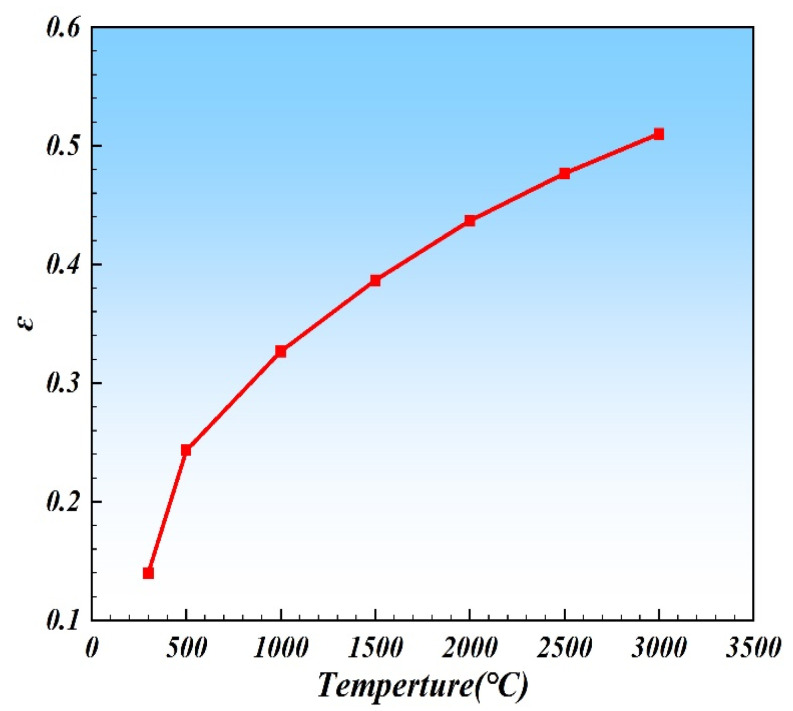
The relationship between ε and temperature.

By calculating the variation of $R_{\rho }$ with temperature, the corresponding temperature-dependent $\sigma_{st}$ was obtained. Substituting this value into Equation (26), the relationship between ε and temperature can be obtained, which in turn affected the Fresnel absorption rate. Table 2 listed the calculated values of ε as a function of temperature, and Figure 6 exhibited the relationship between ε and temperature. The electrical resistivity data of 316L stainless steel used in this study are taken from reference.

Based on the result of Table 3 and Figure 6, the relationship between ε and temperature was expressed as the following polynomial:

(30) $$\varepsilon =-2.00\times 10^{-8}T^{2}+1.70\times 10^{-4}T+0.18$$

**Figure 7:**
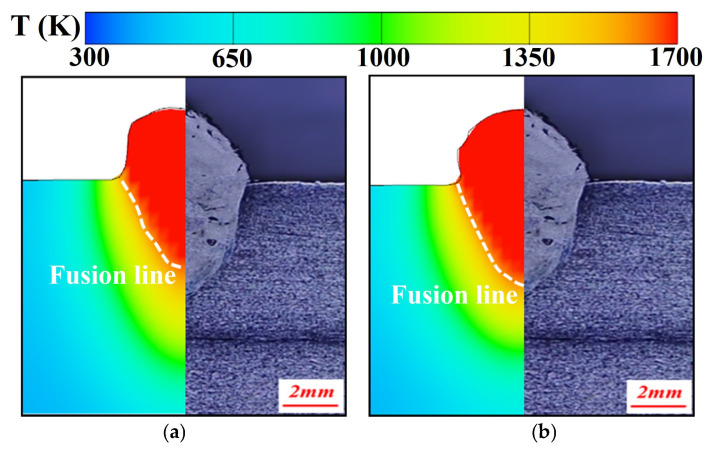
Simulated and experimental weld cross-section comparison in laser deposition: (**a**) before, and (**b**) after optimization.

The above equation indicated that with increasing temperature, ε first increased and then tended to stabilize. Although the quadratic term coefficient was relatively small, it was sufficient to influence the growth rate of ε at high temperatures. To verify the influence of temperature-dependent electrical conductivity, laser cladding simulations were carried out under two different conditions: one with constant electrical conductivity and the other with temperature-dependent electrical conductivity. The simulated cross-sections were compared with those obtained from laser additive experiments, as shown in Figure 7. When the temperature-dependent Fresnel absorptivity was incorporated into the laser heat source model, the numerical results exhibited significantly improved agreement with experimental data (error < 10%), confirming the accuracy and reliability of the optimized model.

**Figure 8:**
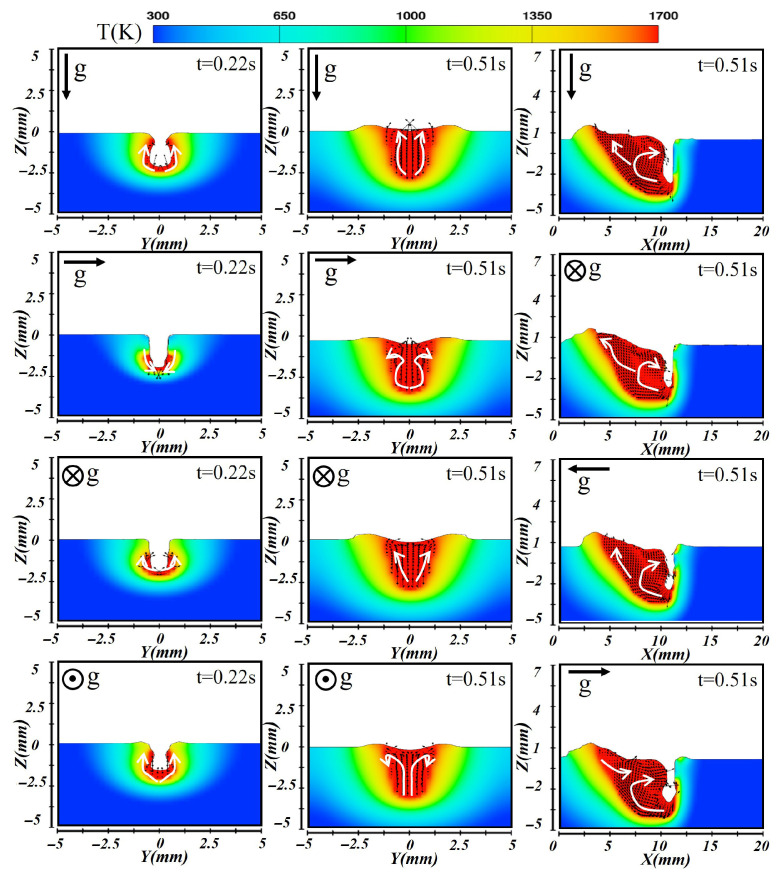
Temperature and flow field distributions of the molten pool cross-section and longitudinal section during laser welding at various spatial orientations.

In addition, numerical simulations of laser wire-filling welding were conducted in four different spatial orientations to obtain the corresponding keyhole morphologies and the distributions of temperature and flow fields, as shown in Figure 8. The white arrows represent the primary flow directions of the molten metal. The results indicated that variations in the gravity direction caused by different spatial positions had little effect on the molten pool shape and internal flow structure, which could be attributed to the dominant role of vapor recoil pressure counteracting the influence of gravity. This finding was consistent with the results reported by Sohail et al. [28]. Therefore, in the simulation of laser–CMT hybrid additive manufacturing, the effect of gravity orientation on laser energy absorption and molten pool dynamics can be reasonably neglected, thereby simplifying the modeling process and improving computational efficiency and numerical stability.

**Table 3:** 316L stainless steel thermophysical properties [29].

Thermophysical Property	Value
Density (kg/m^3^)	7680
Thermal conductivity (W/(m·K))	29.4
Viscosity (kg/(m·s))	5.9 × 10^−3^
Surface tension (N/m)	1.87
Temperature coefficient of surface tension (N/(m·K))	−0.4 × 10^−3^
Specific heat (J/(kg·K))	722
Latent heat of melting (J/kg)	2.6 × 10^5^
Latent heat of vaporization (J/kg)	7.34 × 10^6^
Liquidus temperature (K)	1697.15
Solidus temperature (K)	1674.15
Volume thermal expansion coefficient (1/K)	4.48 × 10^−5^
Heat transfer coefficient (W/(m^2^·K))	1.0 × 10^−8^
Surface radiation coefficient	0.4

#### 2.2.5. Material Properties and Boundary Conditions

The thermophysical properties of 316L stainless steel used in this study were summarized in Table 3.

Initially, the temperature across the domain was fixed at 298 K, and all velocity components were set to zero. The following boundary conditions were applied:

(1) Top surface $A_{2}B_{2}C_{2}D_{2}$

The energy balance on the upper surface was expressed as:

(31) $$-\vec{n}\cdot \left(\lambda \nabla T\right)=q_{s}-{\vec{q}}^{Τ}\vec{1}$$

where $q_{s}$ is the energy source term.

In Equation (31),

(32) $$\vec{q}=\left[\begin{array}{c} \alpha_{c}\left(T-T_{f}\right)\\ \sigma \alpha \left(T^{4}-T_{0}^{4}\right)\\ W_{q}L_{q} \end{array}\right]$$

where $\alpha_{c}$ represents the heat transfer coefficient between the workpiece and the surrounding environment, σ denotes the Stefan-Boltzmann constant, $W_{q}$ is the evaporation rate.

The dynamic boundary condition in the normal direction is given by:

(33) $$-P+2\mu \frac{\partial {\vec{v}}_{n}}{\partial n}=P_{\sigma }-P_{A}-P_{R}$$

where $P_{\sigma }$ stands for the surface tension, $P_{A}$ represents the arc pressure, $P_{R}$ corresponds to the evaporation reaction force.

The tangential momentum boundary condition incorporates the Marangoni shear stress:

(34) $$\mu \frac{\partial {\vec{v}}_{t}}{\partial n}=-\frac{\partial \gamma }{\partial T}\frac{\partial T}{\partial r}$$

where ${\vec{v}}_{t}$ is the tangential velocity vector, ∂γ/∂T is the surface tension gradient, r denotes the tangential direction of the upper surface.

(2) Symmetry plane $AA_{2}D_{2}D$
  (35) $$-\lambda \frac{\partial T}{\partial y}=0,\;v=0,\;\frac{\partial u}{\partial y}=0,\;\frac{\partial w}{\partial y}=0$$

(3) Other surfaces
  Convective and radiative heat losses were considered:
  (36) $$-\vec{n}\cdot \left(\lambda \nabla T\right)=\alpha_{c}\left(T-T_{f}\right)+\sigma \alpha \left(T^{4}-T_{0}^{4}\right)$$

### 2.3. Experimental and Simulation Parameters

To examine how spatial orientation affects the surface morphology in laser cladding, four typical deposition orientations were considered: horizontal, lateral, vertical-down, and vertical-up. The laser–CMT hybrid additive manufacturing simulations were conducted using a scanning speed of 0.36 m/min, a interlayer spacing of 4 mm, and a defocusing distance of 0 mm. In the first layer, high laser power (2000 W) and wire feed speed (6.7 m/min) were applied to stabilize the keyhole, achieve deep penetration, and ensure full substrate fusion. For subsequent layers, to prevent over-melting of the underlying material and interlayer collapse, a conduction-mode heat transfer approach with reduced laser power (1500 W) and lower wire feed speed (5.3 m/min) was employed. This strategy maintained a stable molten pool shape, uniform heating of the underlying material, and consistent deposition quality across layers.

## 3. Results and Discussion

### 3.1. Molten Pool Dynamic Behavior in Horizontal Orientation

**Figure 9:**
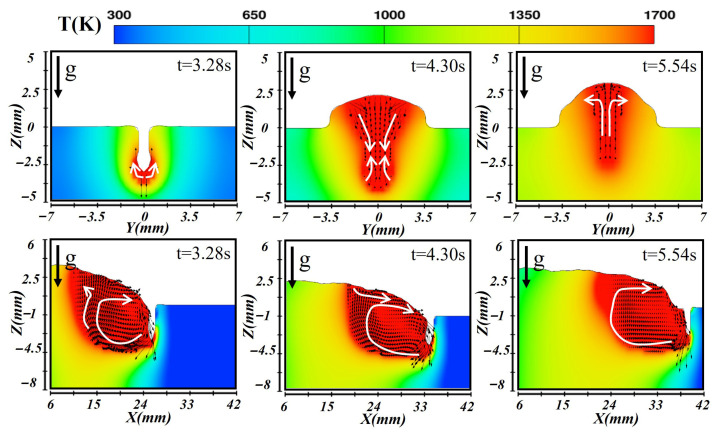
Temperature and velocity distributions of the first-layer molten pool during horizontal laser–CMT additive manufacturing.

**Figure 10:**
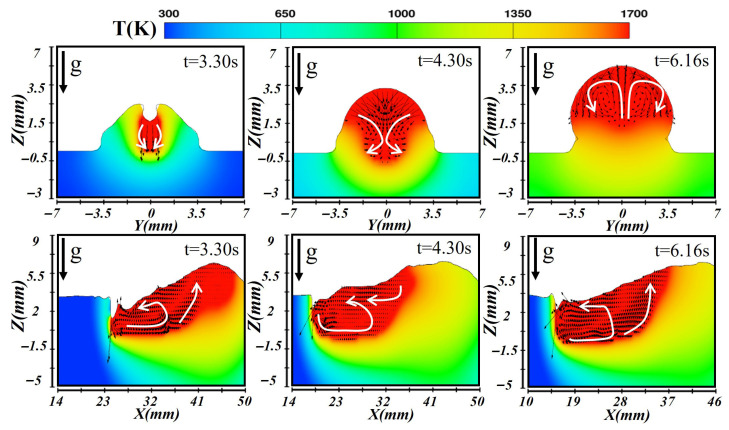
Temperature and velocity distributions of the second-layer molten pool during horizontal laser–CMT additive manufacturing.

**Figure 11:**
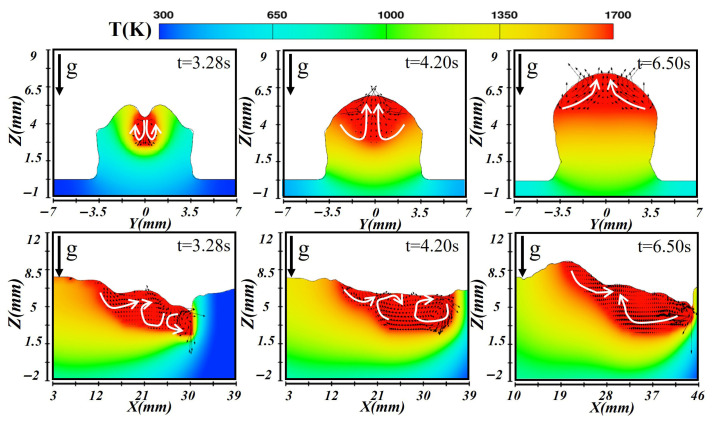
Temperature and velocity distributions of the third-layer molten pool during horizontal laser–CMT additive manufacturing.

Figure 9, Figure 10 and Figure 11 illustrate the simulated temperature and flow fields of the molten pool in both cross-sectional and longitudinal planes during laser–CMT hybrid additive manufacturing under the horizontal orientation. As depicted in Figure 9, during the formation of the first-layer molten pool, the high-energy-density laser induced intense evaporation at the substrate surface, and the resulting vapor recoil pressure drove the molten metal at the molten pool bottom to flow outward, forming a keyhole structure at t = 3.28 s. The temperature at the molten pool bottom was extremely high, and metal near the boundary flowed laterally outward. In the longitudinal section, two distinct flow patterns were observed: liquid metal along the keyhole wall flowed downward under the vapor recoil pressure and then turned toward the trailing edge along the solid–liquid interface; simultaneously, liquid metal at the rear of the molten pool flowed forward toward the keyhole under the combined effects of gravity and surface tension, producing vigorous recirculation. When the molten pool reached a quasi-steady state (at t = 4.30 s), a deep molten pool was maintained under the laser beam, and the surface liquid metal spread laterally under the action of the arc, forming the weld width. Upward backflow occurred at the molten pool bottom, and the stirring effect of the arc enhanced lateral convection, establishing a stable molten pool geometry. During first layer deposition, heat conduction into the substrate dominated due to its high thermal conductivity. At t = 5.54 s, the molten pool entered the solidification stage, gradually releasing heat to the surroundings and forming the weld reinforcement. The molten pool shrank progressively as the high-temperature liquid metal migrated from the central region toward the cooler periphery until the flow nearly ceased.

After the first layer had fully solidified, the subsequent layer was deposited under a lower heat input condition. Figure 10 illustrates the corresponding temperature and velocity distributions during the second-layer deposition. At this stage, heat transfer was dominated by conduction, and the absorbed thermal energy remelted the upper portion of the previous layer. Due to the relatively shallow molten pool, a keyhole did not form under the same conditions. The arc provided additional heat input and drove fluid motion in the molten region. The cross-sectional results indicated that only the top of the first layer underwent partial remelting, while the longitudinal view showed that heat accumulates near the molten pool front and gradually diffused backward. More heat was transferred into the underlying layers, slowing the cooling rate and extending the molten pool length. By t = 4.30 s, the molten pool stabilized with a relatively uniform temperature distribution; the isotherms appeared as a wide, shallow ellipse, and the liquid metal circulated from the center toward the bottom, forming a dual-vortex flow pattern. The reduced heat dissipation delayed cooling, and during solidification (at t = 6.16 s), surface tension drove the remaining liquid metal from the center toward the edges, generating a smooth reinforcement and forming a sound metallurgical bond between layers.

Figure 11 presents the simulation results obtained for the third-layer deposition. Since this layer was deposited on the preformed two-layer substrate, the thermal conduction path changed significantly, affecting both melt pool formation and cooling. Upon combined heating by the laser and arc, the heat was transferred downward through conduction into the previous layers, forming a relatively shallow molten pool as observed at t = 3.28 s. The longitudinal section revealed complex fluid behavior: liquid metal at the front flowed backward, forming vortices, while heat dissipation was hindered by the underlying layers, resulting in an extended molten pool. Meanwhile, molten metal near the rear flowed slowly forward under the influence of the temperature gradient. When the molten pool stabilized at t = 4.20 s, the increase in surface tension facilitated sustained internal circulation, which led to more pronounced convection in the cross-section. The longitudinal temperature distribution exhibited a front-high/rear-low pattern. At the solidification stage (t = 6.50 s), the third layer exhibited the slowest cooling rate among all layers. The inward flow of liquid metal from the pool edges contributed to the formation of a smooth and uniform weld bead.

### 3.2. Molten Pool Dynamic Behavior in Transverse Orientation

**Figure 12:**
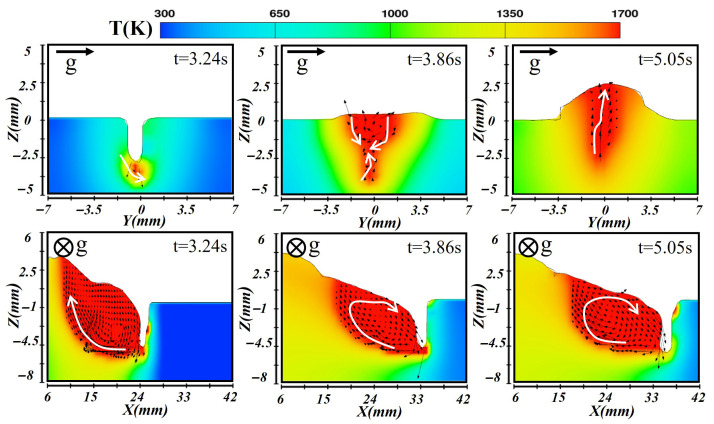
Temperature and velocity distributions of the first-layer molten pool during transverse laser–CMT additive manufacturing.

**Figure 13:**
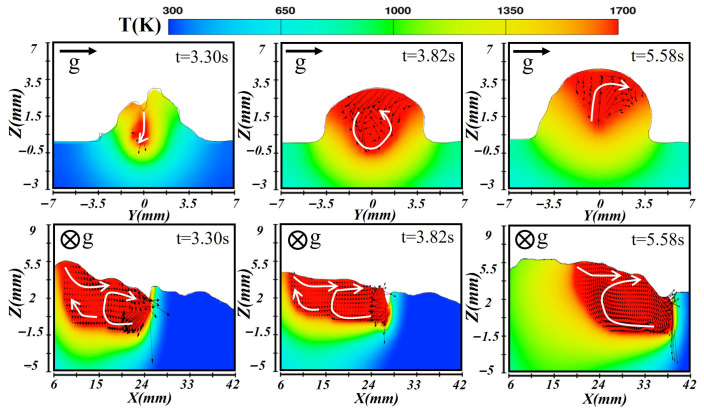
Temperature and velocity distributions of the second-layer molten pool during transverse laser–CMT additive manufacturing.

**Figure 14:**
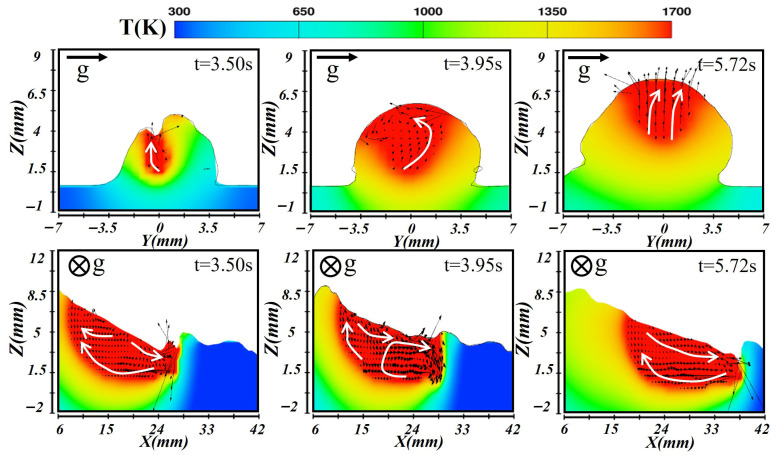
Temperature and velocity distributions of the third-layer molten pool during transverse laser–CMT additive manufacturing.

During transverse deposition, the gravitational force oriented perpendicular to the welding direction notably impacts the molten pool characteristics, as depicted in Figure 12, Figure 13 and Figure 14. At the early stage of molten pool formation, the laser’s high energy density induces a strong vapor recoil pressure, which is typically on the order of 10^3^–10^4^ Pa in the keyhole region. This magnitude is one to two orders higher than the gravitational pressure acting on the molten metal (≈150–300 Pa). As a result, vapor recoil pressure dominates the formation and stabilization of the keyhole, and the change in gravity direction has only a limited effect in this stage. However, the molten metal at the pool bottom tended to flow downward under gravitational force. In the longitudinal plane, the combined action of the laser and arc promoted forward expansion of the molten pool front, while the rear region exhibited relatively slow flow. Once the molten pool reached a quasi-steady state (at t = 3.86 s), the side facing the gravity direction became noticeably wider, leading to an asymmetric shape. The deep keyhole region formed by the laser’s concentrated energy was less affected by gravity, maintaining a dynamic equilibrium. Surface tension resisted complete deformation but sustained the inclination of the molten pool. In the cross-section, convection arose as the high-temperature liquid metal near the bottom flowed upward while the surface layer flowed downward. At the solidification stage (t = 5.05 s), heat rapidly transferred into the substrate, reducing the liquid metal temperature and flow intensity. The remaining molten metal migrated toward the gravity side before solidifying, producing an asymmetric weld cross-section.

For the second layer illustrated in Figure 13, the molten pool transitioned to a heat conduction mode due to the reduced laser power, weakening the laser’s stabilizing effect and amplifying the influence of gravity. At the initial formation stage (at t = 3.30 s), heat was primarily conducted through the top surface into the underlying layer. Gravity caused the molten metal to flow downward, resulting in a thinner upper region, while surface tension drove metal from the hotter to the cooler zones. In the longitudinal section, the front maintained the highest temperature. Once the pool stabilizes (at t = 3.82 s), the combined heat input from the laser and arc produced a relatively steady molten pool. A vortex directed toward the gravity side formed in the cross-section, leading to molten metal accumulation at the lower region. The longitudinal view revealed a high-temperature front and rapidly cooled tail, with arc forced dominating metal flow. During solidification (at t = 5.58 s), the gravity side cooled more slowly, initiating solidification from the bottom upward. Molten metal migrated toward the gravity side, where it finally solidifies, producing an irregular weld shape as shown.

During the deposition of the third layer, the molten pool formed atop the earlier layers, with changed heat conduction dynamics impacting its formation and cooling process, as shown in Figure 14. At the beginning of t = 3.50 s, the laser energy was concentrated at the center, yielding higher temperatures in the central region and a relatively shallow pool due to rapid heat diffusion. In the longitudinal plane, the coupled laser–arc action produced a clear temperature gradient, with the front region significantly hotter than the rear, driving liquid metal from the high-temperature front toward the cooler rear. As heating continues, the pool stabilized (at t = 3.95 s), but molten metal continued to flow toward the gravity side, increasing the weld’s inclination and deposition thickness. The molten pool bottom exhibited relatively uniform temperature, while the top remained hotter. A vortex formed near the front high-temperature zone, while the accumulated rear metal flows forward. During solidification (at t = 5.72 s), the molten pool solidified from the bottom upward, and under gravity, metal further deposited on the gravity side. The opposite side solidified first, and the cooling rate difference ultimately produced an asymmetric, stratified weld structure.

### 3.3. Molten Pool Dynamic Behavior in Vertical-Up Orientation

**Figure 15:**
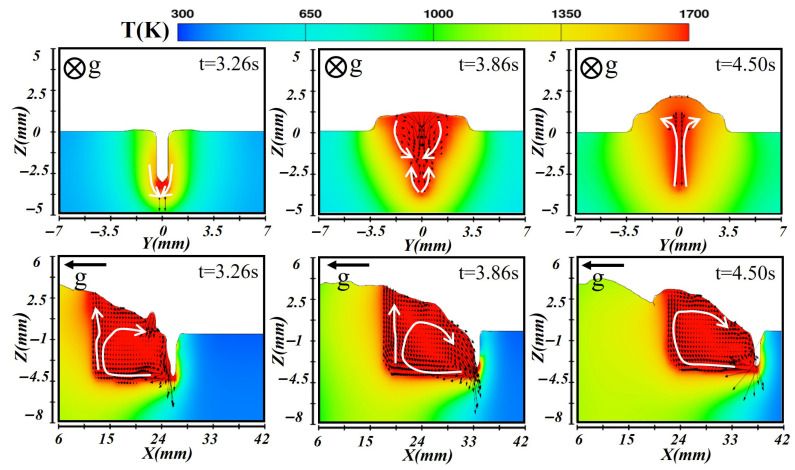
Temperature and velocity distributions of the first-layer molten pool during vertical-up laser–CMT additive manufacturing.

**Figure 16:**
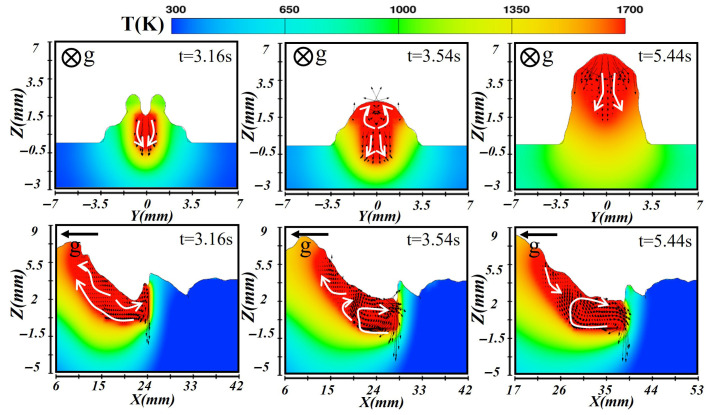
Temperature and velocity distributions of the second-layer molten pool during vertical-up laser–CMT additive manufacturing.

**Figure 17:**
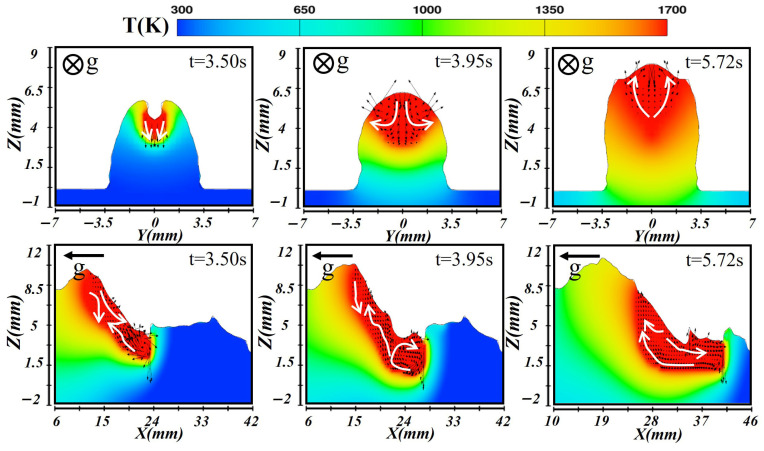
Temperature and velocity distributions of the third-layer molten pool during vertical-up laser–CMT additive manufacturing.

In the vertical-up laser–CMT hybrid additive manufacturing process, the gravity direction was opposite to the deposition direction. Consequently, the molten pool experienced a pronounced gravitational effect during its formation, stabilization, and solidification stages. This effect caused notable variations in temperature distribution, flow patterns, and final bead morphology compared to horizontal or transverse deposition orientations, as illustrated in Figure 15, Figure 16 and Figure 17. Figure 15 shows the simulated temperature and flow fields during the deposition of the first layer. At this stage, a higher laser power was employed. In the initial formation stage (at t = 3.26 s), the molten pool exhibited a greater depth, and a stable keyhole mode was established. The laser’s high energy density generated a localized high-temperature region at the pool center. Along the longitudinal section, the temperature peaked near at the leading edge and gradually decreased toward the rear, forming a stable temperature gradient. Because gravity acted in the opposite direction to deposition, the high-temperature molten metal tended to accumulate toward the rear of the pool. The arc force and surface tension counteracted this gravitational effect, inducing vortex motion and backward flow of the molten metal. When the molten pool reached a quasi-steady state at t = 3.86 s, gravity prevented sufficient lateral spreading, leading to a narrower molten pool shape. The Marangoni effect dominated the molten pool convection, maintaining a stable circulation pattern. The combined heat input from the laser and arc produced a relatively uniform temperature field. During solidification (at t = 4.50 s), the molten pool edges solidified first due to gravitational influence, while the liquid metal inside flowed upward toward the molten pool top, forming a narrow bead with limited width.

Figure 16 illustrates the simulation results of the second layer. With a slightly reduced laser power and total heat input, the molten pool transitioned from a stable keyhole mode to a heat-conduction mode. At the initial formation stage (at t = 3.16 s), laser energy was mainly absorbed at the surface, where metal melting occurred predominantly through heat conduction, leading to a shallow pool. The center of the pool maintained the highest temperature, while the edges were relatively cooler. In the longitudinal section, the front exhibits higher temperatures but shallower penetration. Under the influence of gravity, molten metal accumulated near the weld bottom and flowed backward, resulting in a raised rear pool shape. Once the pool stabilized at t = 3.54 s, the temperature at the center remained high, and thermal conduction yielded a more uniform temperature distribution. The molten metal developed a stable bidirectional flow pattern toward its top and bottom boundaries. In the longitudinal plane, small vortices formed near the front, while liquid metal accumulated at the rear due to gravity. During solidification at t = 5.44 s, the bottom region solidified first. As more heat transferred to the underlying layer, the cooling rate became slower than that of the first layer. The metal flow gradually ceases, and under the influence of gravity, a bead with smaller width but higher reinforcement was formed.

Figure 17 shows the simulated results for the third layer, built upon the previous two layers. At this stage, the molten pool dynamics were governed by accumulated heat, pool stability, and the gravitational effect on molten metal flow. The heat-conduction mode dominates, resulting in a relatively shallow pool in which melting occurred mainly through thermal conduction. During the formation stage at t = 3.50 s, laser energy was concentrated on the pool surface, while the CMT arc provided additional heating. The temperature distribution featured a central high-temperature zone with cooler edges. Along the longitudinal section, gravity drove molten metal downward, causing metal accumulation at the lower side and forming a raised rear bead. When the molten pool stabilized at t = 3.95 s, the cross-sectional flow pattern showed molten metal moving outward from the hot center toward the cooler boundaries. Under surface tension and gravity, molten metal accumulated at the lower region, shaping the new weld bead. During solidification at t = 5.72 s, solidification proceeded gradually from the bottom upward. The rear region solidified first, while the upper portion cooled more slowly. Due to the accumulated heat from previous layers, the third layer exhibited the slowest cooling rate and an extended solidification duration, eventually forming a uniform and well-shaped weld bead.

### 3.4. Molten Pool Dynamic Behavior in Vertical-Down Orientation

**Figure 18:**
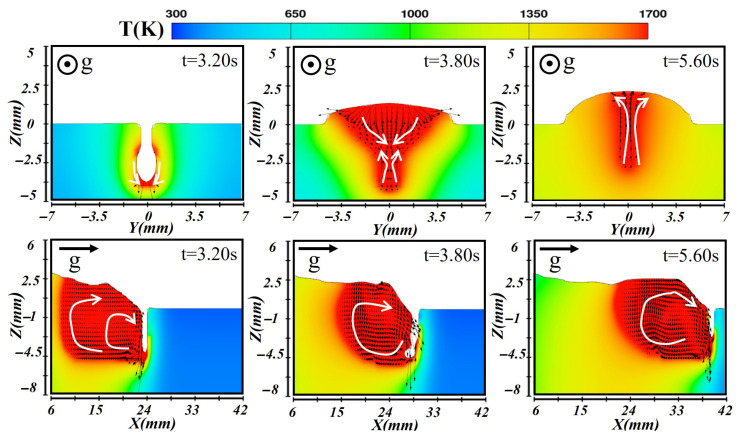
Temperature and velocity distributions of the first-layer molten pool during vertical-down laser–CMT additive manufacturing.

In the vertical-down orientation of the laser–CMT hybrid additive manufacturing process, gravity aligned with deposition direction, greatly promoting the spreading of the molten pool. During the first-layer deposition, the relatively high heat input and laser–arc interaction result in increased penetration, which strongly affected the molten pool dynamics as presented in Figure 18. At the initial formation stage (at t = 3.20 s), the high laser energy density induces rapid metal evaporation, generating vapor-recoil pressure on the order of 10^3^–10^4^ Pa. This magnitude is significantly higher than the gravitational pressure acting on the molten pool, and therefore vapor recoil continues to dominate keyhole formation even in the vertical-down orientation. However, unlike the vertical-up case in Figure 15, gravity acts synergistically with vapor recoil and arc pressure, promoting the downward flow of liquid metal and enhancing flow stability. Most molten metal is transported downward, leading to a more elongated and directionally biased pool shape. When the pool attains stability (at t = 3.80 s), gravity facilitated lateral spreading along the pool width, resulting in a wider bead. Cross-sectional flow shows the bottom liquid metal rising due to recoil pressure, while the surface liquid metal flowed downward under arc pressure. The reduced temperature gradient within the pool established a stable molten pool morphology. During solidification at t = 5.60 s, heat rapidly conducted into the substrate, forming a wide bead with relatively low reinforcement.

**Figure 19:**
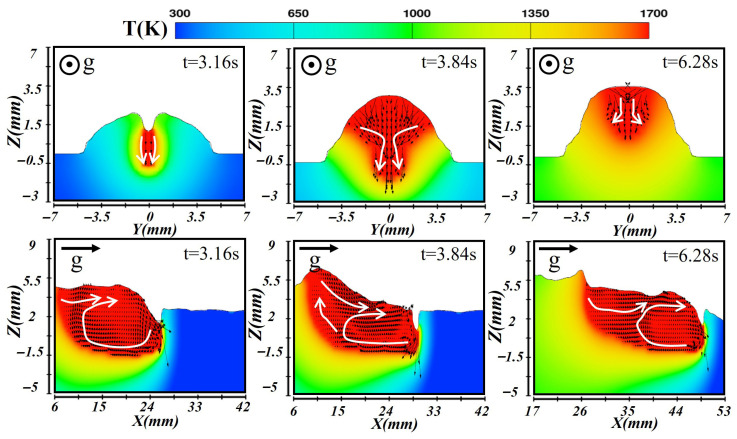
Temperature and velocity distributions of the second-layer molten pool during vertical-down laser–CMT additive manufacturing.

During the second-layer deposition, the heat input was reduced, and the molten pool transitioned from the keyhole mode to the heat-conduction mode, producing a shallower pool incapable of sustaining a stable keyhole as depicted in Figure 19. At the early formation stage of t = 3.16 s, the high laser energy partially remelted the first layer, but the heat-conduction mode limited penetration depth. Gravity induced a forward-directed downward flow of molten metal along the longitudinal section. When the pool reached stability at t = 3.84 s, liquid metal spread laterally due to gravity, but the reduced heat input and surface tension slightly limited pool width, forming a uniformly shaped second-layer bead. During solidification (at t = 5.60 s, heat rapidly conducted into the first layer, accelerating solidification at the pool bottom. The top-layer liquid metal exhibited reduced flow and moved toward the edges. Due to the underlying bead, overall cooling slowed, resulting in a bead width slightly smaller than the first layer.

**Figure 20:**
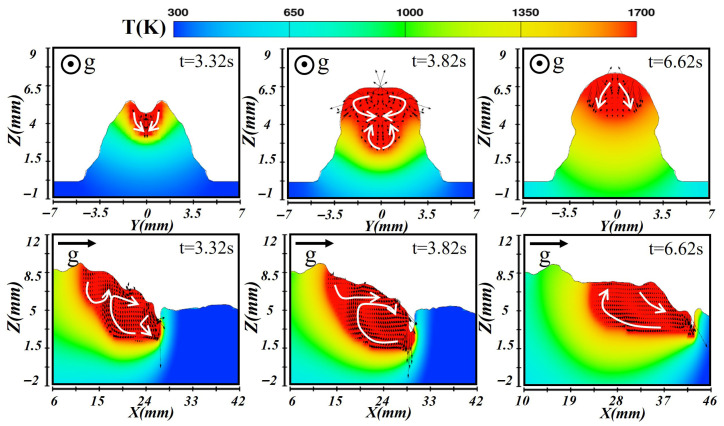
Temperature and velocity distributions of the third-layer molten pool during vertical-down laser–CMT additive manufacturing.

During the deposition of the third layer, the heat-conduction mode continued, and the solidified underlying layers strongly influenced heat dissipation, molten pool flow, and temperature distribution as illustrated in Figure 20. At t = 3.32 s during initial formation, laser heating partially remelted the second-layer bead. With reduced heat input, energy primarily diffused through conduction, producing a shallow pool that avoided instability caused by excessive penetration. In the longitudinal section, high temperatures at the front induced vortical flow, while gravity drove the rear liquid metal forward, with arc and gravitational forces ensuring uniform metal distribution. Once the pool stabilized (at t = 3.82 s), the cross-section showed a relatively uniform temperature distribution: the top high-temperature metal flowed toward the edges, and bottom liquid metal moved upward, maintaining stable convection and forming a uniform bead over the first two layers. During solidification at t = 6.62 s, gravity drove more liquid metal forward, while the influence of the underlying layers slowed heat conduction to the bottom. The third layer exhibited the slowest cooling rate, ultimately forming a wider bead compared with vertical-up deposition, reflecting significant effects from gravity and heat accumulation.

### 3.5. Model Validation

**Figure 21:**
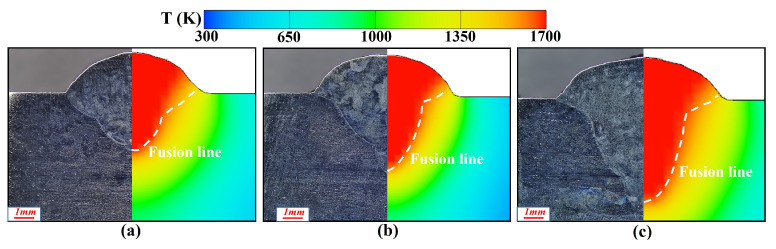
Comparison of simulated and experimental weld cross-sectional profiles of at varying laser powers: (**a**) 1500 W, (**b**) 2000 W, (**c**) 2500 W.

To verify the simulation results, calculated bead cross-sections were contrasted with experimental data. Figure 21 displays the comparison between simulation and experiment for laser–CMT hybrid additive manufacturing across multiple laser power conditions. The results exhibited good agreement, with the predicted fusion line morphology and trends exhibiting errors within the acceptable engineering range (<10%).

**Figure 22:**
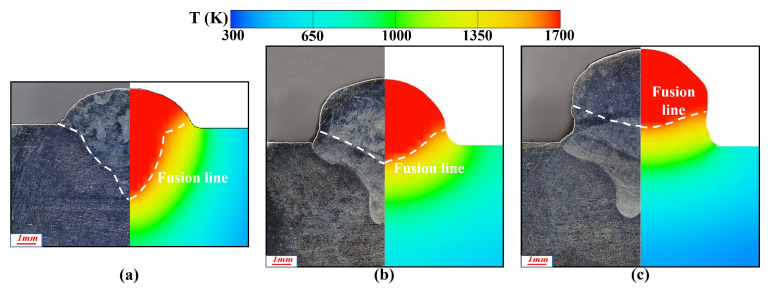
Comparison of simulated and experimental weld cross-section profiles for different layers: (**a**) first layer, (**b**) second layer, (**c**) third layer.

The comparison between simulated and experimental cross-sections of multilayer hybrid deposition is illustrated in Figure 22. The simulation accurately reproduced the molten pool geometry, including width, depth, and reinforcement, demonstrating high consistency with experimental measurements. These comparisons confirmed the accuracy of the established hybrid heat source model and the reliability of the computational method, offering a solid theoretical foundation for the numerical simulation of the laser–CMT hybrid additive manufacturing process.

## 4. Conclusions

This study explored process control in 316L stainless steel laser–CMT hybrid additive manufacturing, focusing on the influence of spatial position on weld bead morphology, molten pool temperature distribution, and fluid flow. The underlying mechanisms of spatial orientation effects on molten pool behavior are elucidated. The key conclusions are presented below:

(1) Numerical simulations revealed that spatial orientation significantly influenced molten pool temperature distribution and flow behavior. In transverse deposition, molten metal migrated toward the gravity side; in vertical-up deposition, gravity limited lateral spreading and increased bead reinforcement; while in vertical-down deposition, gravity enhanced lateral flow but reduced penetration depth. Multi-layer deposition further enlarged the molten pool volume due to heat accumulation and slower cooling.
(2) Despite the variation in orientation, gravity had a limited impact on overall molten pool morphology in laser–CMT hybrid additive manufacturing. The keyhole formation and internal convection were primarily governed by laser power, vapor recoil pressure, and arc-induced forces, with spatial position exerting only minor influence on the global temperature and flow fields.
(3) During layer-by-layer deposition, the molten pool evolved from a keyhole-dominated mode at higher laser power (2000 W) in the first layer to a heat-conduction-dominated mode at reduced power (1500 W) in subsequent layers. The accumulated heat in multi-layer buildup effectively slowed the cooling rate, ensuring stable interlayer bonding and improved bead uniformity.
(4) The simulations demonstrated that variations in scanning speed, wire feed spacing, and defocusing distance jointly influenced bead morphology, temperature gradients, and internal flow structures. These findings provide a reliable numerical basis for optimizing process parameters and developing adaptive heat source models for high-precision laser–CMT hybrid additive manufacturing.
(5) From an engineering perspective, starting with a laser power of 2000 W and a wire feed speed of 6.7 m/min ensures stable keyhole formation and deep penetration in the first layer. Reducing the laser power to 1500 W and wire feed speed to 5.3 m/min in subsequent layers maintains a stable conduction mode, preventing overheating and interlayer defects. With a scanning speed of 0.36 m/min, 4 mm wire feed spacing, and zero defocusing, these parameters balance heat input and layer uniformity, leading to consistent bead formation and improved build quality in multilayer deposition.

## Figures and Tables

**Table 1:** Chemical composition of 316L stainless steel and ER316L welding wire (wt%).

Process Parameter	Value
Laser power (kw)	1.5~2.1
Wire feeding speed (m/min)	4.2~6.7
Welding current (A)	150~210
Scanning speed (m/min)	0.24~0.48
Horizontal position (θ)	−90~90
Vertical-up position (θ)	−90~0
Vertical-down position (θ)	0~90

**Table 2:** Relationship between resistivity, conductivity, laser absorptivity, and temperature.

Temperature (K)	300	500	1000	1500	2000	2500	3000
Electrical resistivity (Ω·m)	7.52 × 10^−7^	2.25 × 10^−6^	3.75 × 10^−6^	5.25 × 10^−6^	6.75 × 10^−6^	8.25 × 10^−6^	9.75 × 10^−6^
Electrical conductivity (S/m)	1.33 × 10^6^	4.44 × 10^5^	2.67 × 10^5^	1.90 × 10^5^	1.48 × 10^5^	1.21 × 10^5^	1.03 × 10^5^
$\varepsilon^{2}$	0.02	0.07	0.11	0.15	0.19	0.23	0.26
ε	0.14	0.26	0.33	0.39	0.44	0.48	0.51

## Data Availability

The original contributions presented in this study are included in the article. Further inquiries can be directed to the corresponding author.
